# Conserved Blood Transcriptome Patterns Highlight microRNA and Hub Gene Drivers of Neurodegeneration

**DOI:** 10.3390/genes16101178

**Published:** 2025-10-10

**Authors:** Jhyme Lou O. De La Cerna, Nicholas Dale D. Talubo, Brian Harvey Avanceña Villanueva, Po-Wei Tsai, Lemmuel L. Tayo

**Affiliations:** 1 School of Chemical, Biological, and Materials Engineering and Sciences, Mapúa University, Manila 1002, Philippines; jlodelacerna@mymail.mapua.edu.ph (J.L.O.D.L.C.); nddtalubo@mymail.mapua.edu.ph (N.D.D.T.); 2 School of Graduate Studies, Mapúa University, Manila 1002, Philippines; 3 International Degree Program in Animal Vaccine Technology, International College, National Pingtung University of Science and Technology, Pingtung 912, Taiwan; j11285355@mail.npust.edu.tw; 4 Department of Food Science, National Taiwan Ocean University, Keelung 202, Taiwan; powei@mail.ntou.edu.tw; 5 Department of Biology, School of Health Sciences, Mapúa University, Makati 1203, Philippines

**Keywords:** neurodegenerative diseases, transcriptomics, neurobiology of brain disorders, hub genes, microRNA

## Abstract

**Background/Objectives**: Neurodegenerative diseases (NDs) such as Alzheimer’s (AD), Parkinson’s (PD), Huntington’s (HD), and Amyotrophic Lateral Sclerosis (ALS) are clinically distinct but share overlapping molecular mechanisms. **Methods:** To identify conserved systemic signatures, we analyzed blood RNA-Seq datasets using Weighted Gene Co-Expression Network Analysis (WGCNA), differential expression, pathway enrichment, and miRNA–mRNA network mapping. **Results:** Two modules, the red and turquoise, showed strong preservation across diseases. The red module was enriched for cytoskeletal and metabolic regulation, while the turquoise module involved immune, stress-response, and proteostatic pathways. **Discussion:** Key hub genes, such as HMGCR, ACTR2, MYD88, PTEN, EP300, and regulatory miRNAs like miR-29, miR-132, and miR-146a, formed interconnected networks reflecting shared molecular vulnerabilities. The absence of classical heat shock proteins in preserved blood modules highlights tissue-specific expression differences between blood and neural systems. Several hub genes overlap with known pharmacological targets, suggesting potential in translational relevance. **Conclusions:** Together, these findings reveal conserved blood-based transcriptional modules that suggest parallel central neurodegenerative processes and may support future biomarker development and possible therapeutic exploration.

## 1. Introduction

Neurodegenerative diseases (NDs) are progressive disorders characterized by the loss of neurons in the brain and spinal cord, leading to cognitive, behavioral, and motor impairments [1]. Globally, NDs impose a profound health risk and socioeconomic burden on affected individuals and their families [2]. Rather than being driven by a single cause, neurodegeneration arises from multiple interacting mechanisms, including mitochondrial dysfunction, oxidative stress, protein aggregation, synaptic loss, neuroinflammation, and disrupted cellular homeostasis [1,3]. These pathological cascades contribute to neuronal injury and death, manifesting as distinct clinical syndromes influenced by genetic predisposition, environmental factors, age, and sex. Currently, ongoing research into neurodegenerative diseases has suggested that these diseases may share molecular and cellular markers, raising the possibility of common transcriptomic signatures underlying their pathogenesis and progression [4].

Among the most prevalent NDs are Alzheimer’s Disease (AD), Amyotrophic Lateral Sclerosis (ALS), Parkinson’s Disease (PD), and Huntington’s Disease (HD). Focusing on individual diseases, Parkinson’s (PD) is a synucleinopathy that is primarily driven by the degeneration of dopaminergic neurons in the substantia nigra pars compacta, resulting in uncontrolled motor movements, affecting individuals with bradykinesia, change in rigidity, and even resting tremors. The well-known pathological signature of PD includes the formation of Lewy bodies and the intracellular aggregates of misfolded α-synuclein, which propagates in a prion-like manner and disrupts proteostasis [5,6]. Aside from motor dysfunction, PD also affects non-motor symptoms such as autonomic dysfunction, REM sleep behavior disorder, and cognitive impairment, reflecting its multisystem nature. While most cases are sporadic, mutation in certain genes may implicate genetic susceptibility, with environmental factors exacerbating disease progression [6,7,8]. Alzheimer’s Disease (AD), which is the common cause of dementia, is an ailment that is defined by extracellular amyloid-beta (Aβ) plaques and intracellular neurofibrillary tangles or NFTs that are composed of hyperphosphorylated tau proteins [9,10]. The pathologies of said ailment can trigger or cause synaptic loss and neurodegeneration, especially in the hippocampus and cortex sections of the brain, leading to progressive memory decline, cognitive impairment, and, in some instances, a personality shift [11]. Furthermore, genetic risk factors like the APOE ε4 allele and mutation could also play a significant role in disrupted protein processing in AD [12].

Amyotrophic Lateral Sclerosis (ALS), which is another neurodegenerative disorder, is characterized by progressive motor neuron degeneration, leading to muscle weakness, paralysis, and in severe cases, respiratory failure. Cytoplasmic TDP-43 aggregates are observed in most ALS cases, whereas familial forms are linked to diverse mutations, causing dysregulation of RNA metabolism and protein homeostasis in disease pathogenesis [13,14,15]. Another ND is Huntington’s Disease, which an illness that is caused by an autosomal disorder caused by CAG trinucleotide expansion in the HTT gene, which leads to a mutation with clinical manifestations such as protein aggregation, striatal neuron loss, psychiatric, and cognitive symptoms [16,17]. Although these NDs exhibit diverse clinical manifestations, they may share convergent pathogenic mechanisms, including proteinopathy, mitochondrial dysfunction, neuroinflammation, and impaired autophagy [4,18].

Uncovering these shared molecular signatures requires transcriptome profiling. RNA Sequencing (or RNA-Seq) has emerged as a powerful tool for capturing gene expression in NDs. While brain tissue is ideal for mechanical insights, blood-based RNA-Seq offers a non-invasive window into systemic and disease-associated molecular changes, with growing evidence supporting its value as a biomarker for neurodegeneration [19,20,21]. To interpret such complex transcriptomic data, Weighted Gene Co-Expression Network Analysis (WGCNA) is utilized in most analyses. WGCNA is a widely used system biology approach that determines or identifies a cluster of genes in a group through modules that are highly correlated in terms of expression patterns across samples. By relating these modules to external traits or phenotypes, WGCNA provides insights into the functional organization of the transcriptome, enabling comprehensive insight into biological pathways and regulatory networks. Implemented as an R package, WGCNA (version 1.73.) constructs a gene co-expression network by calculating pairwise correlations between genes then applying a soft-thresholding power to emphasize strong correlations while dampening noise [22]. Key output includes modules of co-expressed genes and the identification of highly connected “hub” genes, which often serve as critical regulators or biomarkers [23]. In neurodegenerative disease research, WGCNA leverages RNA-Seq data from various repositories to uncover disease-associated modules, revealing conserved and condition-specific transcriptional disruptions. By integrating clinical variables, the analysis further dissects how these factors shape network architecture and contribute to disease heterogeneity. This approach not only deciphers complex transcriptomic changes but also facilitates the discovery of therapeutic targets and precision medicine strategies [24].

Forming another important regulatory layer, MicroRNAs (miRNAs) are critical post-transcriptional regulators that fine-tune gene expression by binding to messenger RNAs (mRNA) and modulating their stability or translation. Through this layer of control, miRNAs can simultaneously regulate multiple targets within a network, amplifying or dampening entire biological pathways [25,26]. In neurodegenerative diseases, dysregulated miRNAs have been linked to synaptic dysfunction, protein aggregation, metabolic imbalance, and neuroinflammation. This is because a single miRNA can target multiple mRNAs, and conversely, each mRNA can also be regulated by multiple miRNAs; these interactions form dense regulatory networks that may amplify biological effects and create points of convergence across distinct disease mechanisms [27].

Mapping miRNA–gene networks within preserved co-expression modules allows us to move beyond descriptive clustering towards a mechanistic understanding of shared molecular signatures in ND. We specifically focused on AD, ALS, HD, and PD because these disorders, while clinically distinct, exhibit overlapping pathological mechanisms such as protein aggregation, neuronal loss, and dysregulated gene expression [4,28]. Moreover, by integrating interactions of hub genes from NDs, we can identify the regulatory axes that are triggered. This system-level approach highlights miRNA not only as upstream regulators of gene modules but also as potential biomarkers and therapeutic targets capable of restoring network balance across multiple NDs.

In this study, we analyzed conserved gene co-expression modules from whole-blood transcriptomic profiles of AD, ALS, HD, and PD individuals. RNA-Seq datasets were retrieved from the Gene Expression Omnibus (GEO) and processed under strict criteria. WGCNA was applied to construct disease-specific networks and identify modules preserved across conditions. To establish biological significance, we performed functional annotation, pathway enrichment analysis, differential expression analysis, and protein–protein interaction (PPI) network characterization. Finally, we integrated microRNA–gene interactions to uncover regulatory axes to highlight hub genes and miRNA as potential biomarkers and therapeutic targets in neurodegeneration.

## 2. Materials and Methods

### 2.1. Acquisition of Dataset

Transcriptomic datasets are publicly available and were retrieved from the Gene Expression Omnibus (https://www.ncbi.nlm.nih.gov/geo/ accessed on 10 July 2025) (GEO) database [29]. The selected datasets include the following, as seen in Table 1.

To ensure biological relevance and comparability, the dataset must be of human (Homo sapiens) origin, from RNA-Sequencing platforms, and must include experiment types such as Expression Profiling by High-Throughput Sequencing and must be from blood. Demographic details can be seen in Table S1. Although some datasets have sample sizes that are relatively small (PD, *n* = 26), prior studies have demonstrated that robust gene co-expression networks analyses can be conducted with a limited sample number using methods such as WGCNA and DESeq2, which account for variability and are effective for small datasets [34,35].

### 2.2. Data Preprocessing and Quality Control

Gene expression data were first filtered to include only genes expressed in at least 20% of samples, ensuring biological relevance. The retained genes were normalized using variance stabilizing transformation (VST), a method that reduces technical noise and makes the variance independent of the expression level. An R function is implemented in DESeq2 package, which reduces heteroscedasticity while preserving between-sample variance. The design formula incorporated available covariates such as age and sex when present in the dataset metadata (AD and HD Datasets contain covariates), thereby reducing confounding effects on expression variance. When covariate information was unavailable, normalization proceeded under a design ~1 to maintain consistency across datasets. Covariate adjustment was further refined by regressing out the effects of age and sex before performing the WGCNA using the limma’s removeBatchEffect function for datasets containing this information, ensuring harmonized gene expression across heterogenous sources [36].

For the AD dataset (GSE249477), subjects clinically diagnosed with mild cognitive impairment (MCI) were grouped alongside the disease category to reflect the biological continuum between MCI and Alzheimer’s disease, which has been demonstrated in other transcriptomic studies. In a study by Huang et al. [37], it was determined that many gene co-expression modules are preserved between MCI and AD blood transcriptomes. They share overlapping transcriptomic signatures, especially in immune activation oxidative stress and synaptic dysfunction pathways. In another study, using transcriptomic-based machine learning, it was determined that biomarkers in MCI were significantly associated with the progression to AD [38]. This grouping also ensured sufficient statistical power for robust co-expression network construction and downstream preservation analysis [39,40].

### 2.3. Weighted Gene Co-Expression Network Analysis (WGCNA)

Utilizing the application R version 4.4.1, we applied Weighted Gene Co-Expression Analysis (or WGCNA) and relevant packages from Bioconductor 3.21 to determine the co-expression patterns and identify conserved transcription modules across neurodegenerative diseases. The analysis was conducted independently for AD, HD, PD, and ALS to allow for network-based comparison and module preservation assessment across datasets.

#### 2.3.1. Network Construction and Scale-Free Topology Estimation

The filtered, normalized data were then evaluated for compliance with the scale-free topology criterion. Using the pickSoftThreshold, we calculated the fit of the overall network to create a scale-free model across soft-thresholding powers (β). The soft-thresholding power is chosen to emphasize strong gene–gene correlation while penalizing weak, potentially spurious ones, thereby boosting the network’s conformity to a biologically relevant scale-free topology. For each dataset, the power value achieving a high-scale free topology model fit (R2) was selected. The dataset with the highest R2 was designed as the reference network for downstream preservation analysis [41,42].

#### 2.3.2. Module Detection and Topological Overlap-Based Clustering

Using the selected soft-thresholding power, we constructed the adjacency matrix through the adjacency function and transformed it into a TOM or a topological matrix, which captures both direct and indirect gene connections for more reliable clustering. Hierarchical clustering of the TOM-identified gene modules was defined with a minimum size of 30 genes [43,44]. For each module, an eigengene summarizes its overall expression profile. Module membership (kME) was then calculated using Pearson’s correlation between each gene and its module eigengene, allowing us to rank genes and identify highly connected hub genes [45].

#### 2.3.3. Cross-Dataset Evaluation of Module Preservation

Module preservation across datasets was evaluated using the module. Preservation function with 100 permutations. To ensure robustness of the module preservation across datasets, it was then rerun and evaluated with 1000 permutations. This approach compared both module density and connectivity structure, with preservation quantified by the composite Z summary statistics [46]. The Z summary threshold used for interpretation is presented in Table 2. Modules with Z summary > 10 were considered strongly preserved, while scores between 2 and 10 indicated moderate preservation, and scores ≤2 indicated no evidence of preservation as seen in Table 2. Only robustly preserved modules (Z summary > 10) were prioritized for downstream analysis as they represent stable co-expression networks conserved across neurodegenerative conditions rather than dataset-specific artifacts.

### 2.4. Function Annotation, Pathway Analysis, and Network Characterization of Modules

Hub genes were identified through a step process beginning with the calculation of module membership (kME) values. Genes from strongly preserved modules were subsequently used to construct protein–protein interaction (PPI) networks using STRING at medium confidence (STRING score ≥ 0.4) [47]. The STRING network was then further analyzed in Cytoscape v3.10.3, where hub genes were identified via the CytoHubba plugin using degree centrality. The top 15 most connected genes per module were selected as key network hubs, representing the most essential connection with each preserved co-expression module [48].

### 2.5. DEA of Hub Genes

To validate the disease relevance of the identified hub genes, while maintaining the integrity of each independent dataset, a targeted differential expression validation was performed. For each of the four neurodegenerative disease datasets, an independent differential expression analysis was run using DESEq2 with the appropriate design formula. From each analysis, the top 3000 most significant genes were extracted. The pre-defined list of 30 hub genes (15 per module) was then manually cross-referenced against these disease-specific top gene lists. A hub gene was considered to have supporting evidence for dysregulation if it was present in the list and met the criteria outlined in our tiered evidence framework, which follows approaches used in transcriptomic studies, which can be seen in Table 3. This strategy confirms that a hub gene is not only central to the co-expression network but also shows consistent transcriptional dysregulation within its original disease context, without the technical confounders of a cross-dataset meta-analysis.

The cutoff of |log_2_FC| ≥ 0.5 was selected to reflect moderate expression shift while avoiding noise from small effect sizes, which is consistent with the previous transcriptomic analysis that emphasizes interpretability over arbitrary stringency [49]. Many transcriptomic papers also utilized said cutoff to determine more biological relevance of genes that may affect shared molecular signature of different ailments [40,50]. This threshold balances statistical sensitivity with biological interpretability, especially in NDs, wherein signatures with large fold changes are uncommon due to cellular heterogeneity and systemic modulation [40,49,50,51,52].

**Table 3:** Tier System of DEGs.

Tier	Criteria	Rationale and Reference
Tier 1—High Confidence	FDR/*p* ≤ 0.05	Commonly used in ND studies, but often misses subtle changes. Utilized by Kurvis et al. [53] and Chen et al. [54] in their papers.
Tier 2—Moderate Confidence	Nominal *p* < 0.05	Bases are on log2FC. It is seen to be utilized in the paper of Salemi et al. [51] and Lai et al. [52].
Tier 3—Cross Disease	Nominal *p* < 0.05 in ≥2 diseases/datasets, regardless of FDR	This reflects the replication across datasets or diseases; it prioritizes consistency over statistical stringency [55]. This is seen as used in the paper of Li et al. [56] and Goodwani et al. [57].

### 2.6. miRNA-Module Regulatory Determination and Enrichment Analysis

miRNA regulators of preserved modules were identified using the multiMiR package, which integrates twelve databases of experimentally validated and predicted interactions. Highly connected genes (kME ≥ 0.6) from the red and turquoise modules were prioritized as targets. Enrichment was assessed with Fisher’s exact test and corrected for multiple testing using FDR. Data were processed with tidyverse, an R package for data wrangling and visualization. Interactions were retrieved with multiMiR, networks were built in igraph, and enrichment analyses (Gene Ontology and KEGG) were performed with the R package, clusterProfiler. Validated interactions were obtained from miRTarBase and TarBase, while prediction came from TargetScan, allowing for the construction of hub gene–miRNA regulatory networks and the identification of axes contributing to neurodegenerative mechanisms.

### 2.7. Integration of Hub Genes with GWAS-Identified Traits

Hub genes identified from the network analysis were annotated for trait and disease association using the Open Targets Platform database (https://platform.opentargets.org/ accessed on 5 October 2025) [58]. For each hub gene, we retrieved reported associations, including GWAS hit, known mutations, pathway involvement, and literature-supported disease links. The retrieved data from the platform were subjected to data processing and analysis using R version 4.4.1. The data processing stage includes the summarization of the number of associated diseases per gene and the identification of diseases (likewise NDs) commonly linked to hub genes. These analyses aimed to provide a structured overview of the genetic association of hub genes with complex traits and diseases.

## 3. Results

### 3.1. WGCNA

#### 3.1.1. Data Pre-Processing and Estimation of Scale-Free Network

Following quality control and normalization, which can be seen in Figure S1, the four datasets—AD, ALS, HD, and PD—were processed to ensure comparability for downstream network analysis. The initial number of detected genes varied, ranging from 13,721 in AD to 25,949 in PD. After filtering for missing values and applying a variance stabilizing transformation (VST), all datasets retained their gene and sample dimensions, which demonstrated adequate data quality and completeness. To reduce the technical noise and emphasize biologically informative patterns, genes common across all datasets were identified, yielding 12,240 shared features. From there, the top 5000 most variable genes were selected using median absolute deviation (MAD) filtering. This step enriched the analysis for genes with the highest variability, thereby increasing the likelihood of capturing the meaning of co-expression structures across diseases with distinct yet overlapping molecular pathologies.

**Figure 1:**
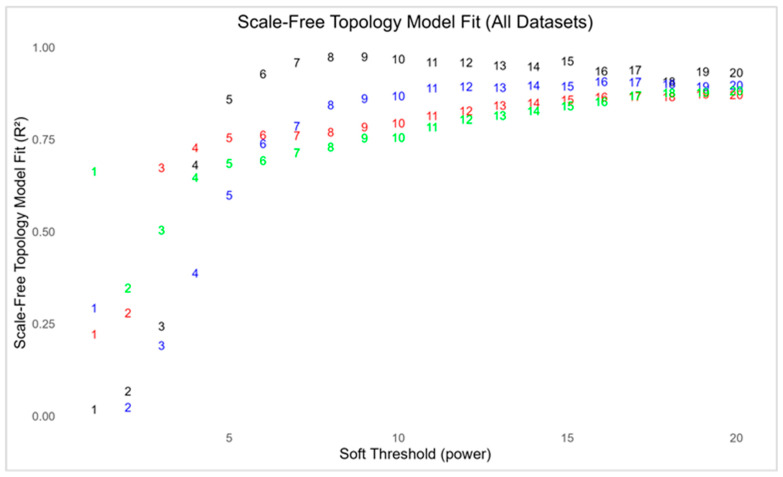
Neurodegenerative diseases scale-free topology model.

The filtered datasets were subjected to scale-free topology (SFT) analysis to evaluate their suitability for WGCNA, as seen in Figure 1. In all four datasets, the network topology approached moderate soft thresholding powers, which is consistent with the biological expectations that gene co-expression networks are non-random at power 6 and hierarchically organized. For AD, the scale-free fit index (R2) exceeded 0.90 at power 6 and stabilized above 0.95 from power 8 onwards, indicating the strongest conformity to scale-free topology. HD required a slightly higher threshold, achieving R2 > 0.85 between powers 13 and 16. PD, on the other hand, showed weaker fits at lower threshold but surpassed R2 = 0.85 at power 10 and maintained stability thereafter. ALS demonstrated similar behavior, with the fit improving gradually and surpassing R2 = 0.85 around powers 9 to 10. The individual SFT evaluation can be seen in Figure S2.

#### 3.1.2. Gene Co-Expression Module Detection Using TOM Similarity

**Figure 2:**
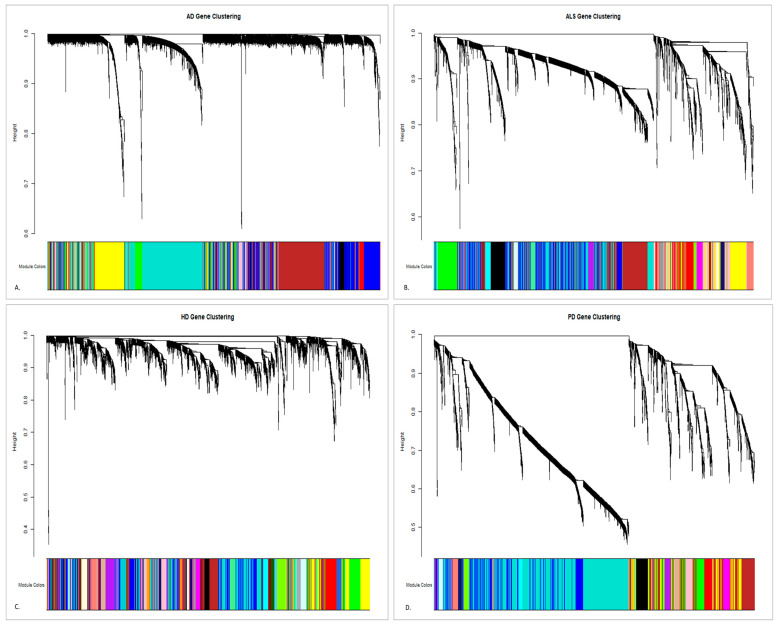
Gene clustering of (**A**) AD, (**B**) ALS, (**C**) HD, and (**D**) PD.

In constructing the TOM-based co-expression networks, AD was designated as the reference due to its strong tendency to scale-free topology, as compared to the higher powers required for HD. This hierarchical clustering dendrogram derived from TOM dissimilarity revealed clear modular structures, which were refined using dynamic tree cutting. Through this algorithm, the parameter is controlled with respect to how finely the tree or dendrogram is split into modules, wherein a lower value results in many smaller modules, while a higher value yields fewer but larger modules. The optimization led to the following results of 0.4–1.0 for HD, 0.5–1.0 for PD, and 0.6–1.0 for ALS, as seen in Figure 2. These different cutting ranges or thresholds directly influenced the resolution of modules, with HD producing the greatest number of smaller, distinct clusters, PD yielding an intermediate distribution, and ALS showing fewer but larger and more cohesive modules. This reflects the underlying network topology, wherein HD displays fragmented co-expression patterns consistent with heterogeneous transcription disruptions, while ALS forms tightly connected TOM clusters, each reflecting a convergent pathogenic pathway.

Altogether, WGCNA identified a total of ten co-expression modules on TOM similarity, each arbitrarily assigned a color: turquoise (1000 genes), gold (1000), blue (993), brown (961), yellow (581), gray (281), green (252), black (252), red (206), and pink (167). The turquoise and gold modules represented the largest TOM clusters, suggesting robust gene-to-gene connectivity and potentially pan-neurodegenerative processes, while the blue and brown modules also formed dense branches in the dendrograms, pointing to stable network communities. In contrast, smaller modules, such as red, black, green, and pink, were detected only at lower cutting thresholds and correspond to highly specific but less interconnected TOM branches. The gray module, with 281 genes, is a representation of unassigned or weakly clustered genes and was less informative for network-level interpretation. These findings emphasize the role of TOM in preserving strong co-expression relationships while also capturing finer modular distinctions across diseases. Large modules reflect stable, highly interconnected gene networks, together providing a structural basis for cross-disease comparison in neurodegenerative disorders.

### 3.2. Module Preservation Analysis

**Figure 3:**
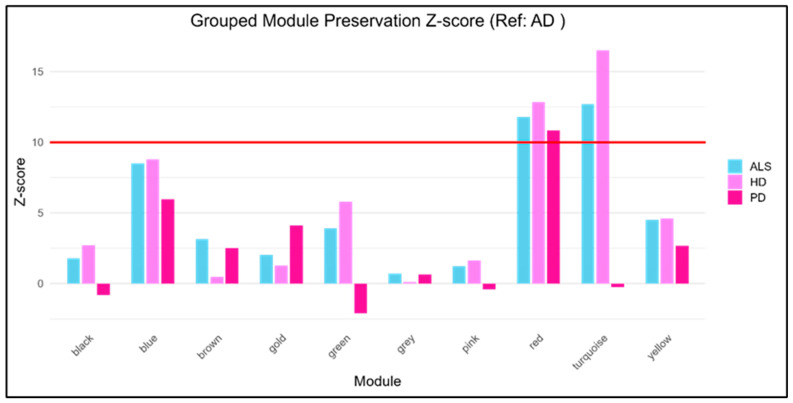
Bar plots of different preserved modules.

Module preservation analysis was conducted with the AD as the reference dataset to evaluate the stability of gene co-expression modules across ALS, HD, and PD. The results show that certain modules, such as the red and turquoise, remained strongly preserved, indicating robust topological overlap and consistent co-expression across disorders, as seen in Figure 3.

The red module, although relatively small in terms of size, demonstrated high reproducibility in all three diseases, suggesting that it may represent fundamental cellular housekeeping functions. The turquoise module, by far the largest cluster, was preserved in HD and ALS but disrupted in PD. While the PD dataset included only 26 samples, as compared to other dataset samples, the reduced size may have limited statistical stability and may have contributed to the weaker preservation signal. Nonetheless, biological contribution is also plausible, pointing to a disease-specific breakdown which may pertain to the mitochondrial and synaptic networks in the dopaminergic system, causing a sudden shift or disruption. This suggests that the disruption is not solely a technical artifact but may reflect genuine disease-related processes [59,60]. Additionally, no modules were uniquely preserved in a single disease dataset. Both the red (206) and turquoise (1000) modules demonstrated strong preservation (Z summary > 10) across different NDs. This indicates that the preserved modules in blood reflect convergent transcriptional programs across NDs rather than disease-specific modules. This can be seen in Table 4 and Dataset S1. Moreover, to maintain robustness, the 1000 permutation rerun does not show drastic change in terms of its Z summary as compared to the 100 permutations. This can be seen in Dataset S1.

### 3.3. DEA, Functional Annotation, and PPI Network of Preserved Gene Co-Expression Modules

The preserved red and turquoise modules were prioritized for downstream analysis. This is because preserved modules have stability, which also offers a stronger basis for identifying hub genes and translational targets since candidate drivers are embedded within reproducible transcriptional programs rather than single-dataset evidence [61,62]. To evaluate the disease relevance of their key regulators, we performed differential expression analysis on the 30 candidate hub genes (15 from each module) and further assessments of biological relevance using the GeneCards database [63] and supporting literatures. Because transcriptomic shifts can be subtle, we applied the tiered differential expression framework seen in Table 2. Consistent fold changes may reflect meaningful biology. By integrating module preservation with this tiered approach, candidate hub genes were prioritized as both embedded in stable co-expression networks and supported by transcriptional dysregulation across diseases [64,65]. Applying our tiered evidence framework, we found that 16 out of 30 hub genes showed evidence of differential expression (meeting Tier 2 or Tier 3) in at least one disease, with the majority of changes observed in the AD dataset, as seen in Table S2.

**Figure 4:**
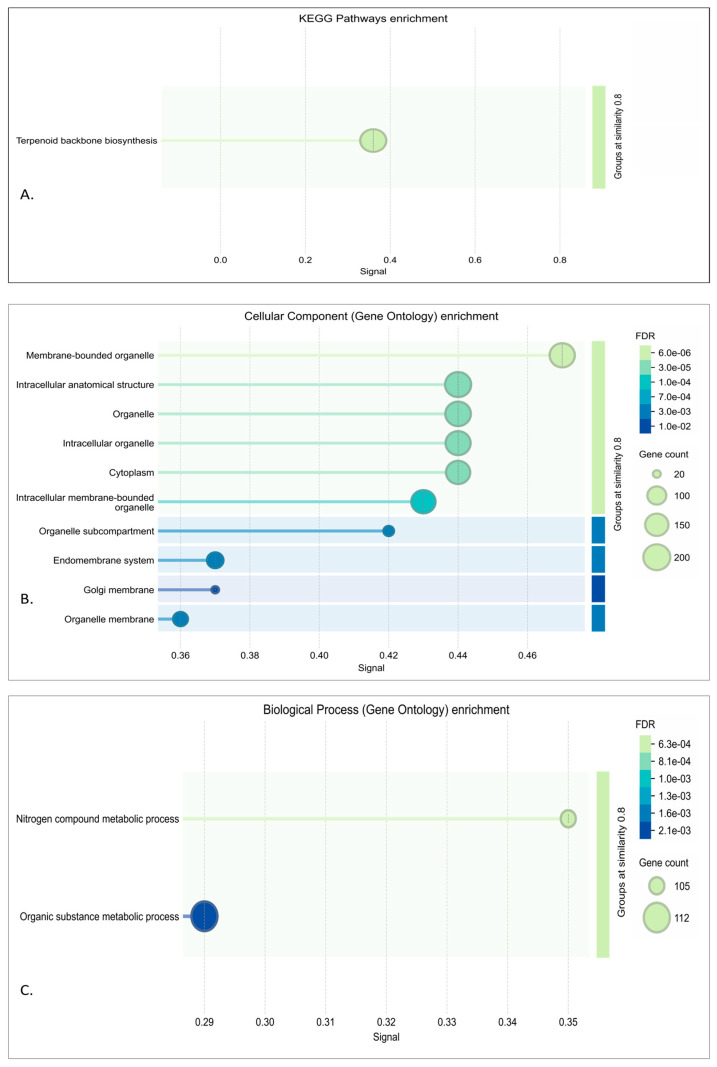
Functional annotation and enrichment analysis of red module: (**A**) KEGG; (**B**) GO CC; (**C**) GO BP.

**Figure 5:**
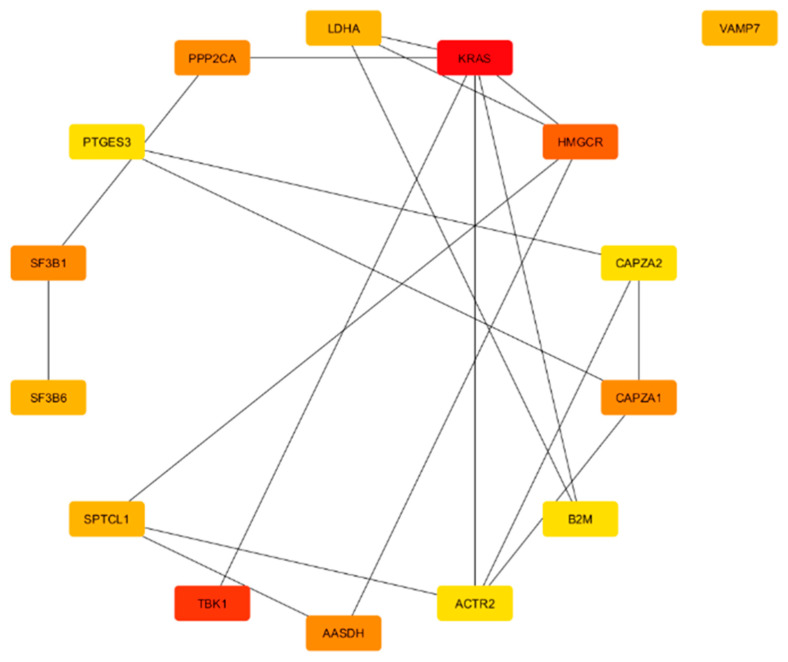
Top 15 hub genes in red module.

**Figure 6:**
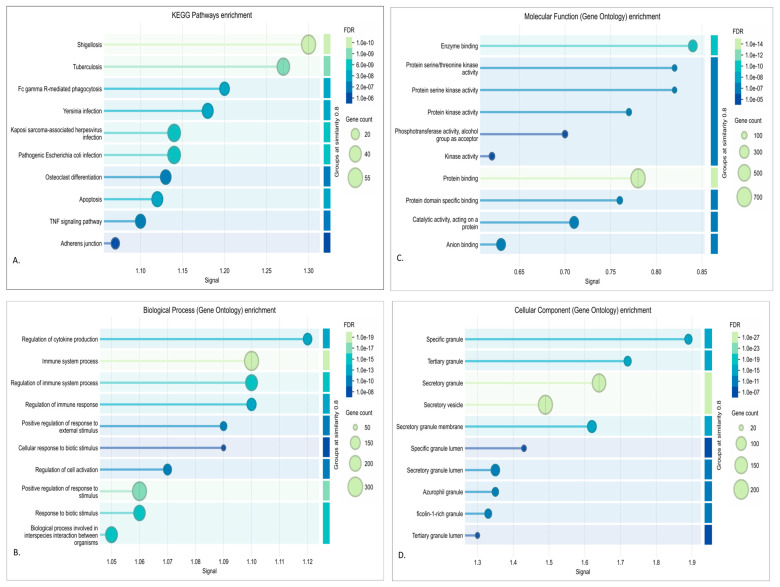
Functional annotation and enrichment analysis of turquoise module: (**A**) KEGG; (**B**) GO BP; (**C**) GO MF; (**D**) GO CC.

**Figure 7:**
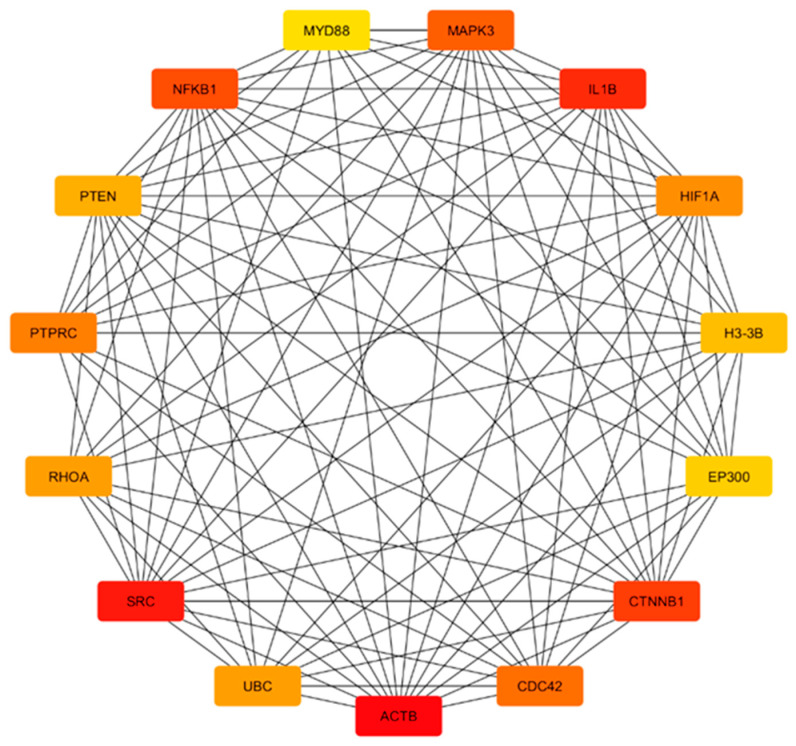
Top 15 hub genes in turquoise module.

From the result of the cytohubba, in terms of degree, the top 15 hub genes, which are seen in the red and turquoise modules, were identified as the key regulators of the network, as seen in Figure 4, Figure 5, Figure 6 and Figure 7. These hub genes represent the structural and functional backbone of the red module as their high connectivity positions them at critical points of intracellular signaling, metabolic regulation, and vesicle transport as seen in Figure 4 and Figure 6. The lines connecting the nodes seen in Figure 5 and Figure 7 are a representation of the interaction links among the identified hub genes, as derived from PPI network analysis. Each line indicates a known or predicted functional association such as direct physical interaction, co-expression, or shared participation in common signaling or metabolic pathways. Moreover, the density of the connecting lines reflects the degree of interaction among genes, where highly interconnected nodes suggest central regulatory roles within the network. In the red module, it can be seen that KRAS and TBK1 exhibited multiple connections, and the same goes for ACTB and PTEN in the turquoise module. This may indicate that these could serve as key integrators, bridging various biological processes relevant to NDs. This integrated prioritization strengthens the hub genes for downstream functional annotation and miRNA–mRNA interaction.

#### 3.3.1. Red Module Result Analysis

In the enrichment analysis results of the red module, it has been shown that its genes are strongly associated with intracellular organization, particularly membrane-bound organelles, cytoplasmic structures, and organelle membranes such as the Golgi and endoplasmic reticulum. This points to a functional role in processes that depend on compartmentalized regulation, including intracellular trafficking and membrane-associated activities. The localization of red module genes within this organelle system suggests that this module captures key elements of the cellular machinery disrupted in neurodegenerative conditions [66]. This can be seen in the functional annotation of Gene Ontology and KEGG in Figure 4.

In the red module, genes such as KRAS, TBK1, and HMGCR exhibit strong interlinkages, which shows a possibility of coordinated activity between lipid metabolism, immune response, and signaling regulation as seen in Figure 5. Moreover, in a biological context, the usage of these genes differs based on their main purpose; for example, VAMP7 is central to vesicle trafficking and synaptic maintenance [67], while HMGCR regulates cholesterol biosynthesis, which is a pathway for neuronal stability [68]. Similarly, KRAS and PPP2CA integrate signaling cascades that connect stress responses to broader regulatory networks [69].

Furthermore, it was determined through differential expression analysis (DEA) that seven hub genes from the module are upregulated and have varying functions in terms of neurodegenerative diseases. The detailed summary of their regulatory direction, biological function, and neurodegenerative relevance is described in the Supplementary Materials (Table S2: Regulation, Table S3: Functional and Neurodegenerative Relevance), which expand upon the literature sources that is further elaborated in the Discussion section [53,70,71,72,73,74,75,76,77,78,79,80]. Additional supporting studies relevant to the molecular mechanism summarized in these tables are also cited in the Supplementary Materials and are included here for completeness [75,76].

#### 3.3.2. Turquoise Module

Enrichment analysis of the turquoise revealed a strong association with immune and stress-response pathways, consistent with a mechanism often implicated in neurodegeneration. Biological process terms were dominated by immune response, positive regulation, and cytokine signaling, highlighting the involvement of glial activation and inflammatory cascades. Molecular function enrichments pointed to protein binding and kinase activity, suggesting the dysregulation of signaling kinases central to tau phosphorylation and synaptic failure. Based on the cellular component enrichment, our study has determined vesicles and secretory granules, indicating disruptions in intracellular trafficking and the lysosomal clearance system. KEGG pathways such as apoptosis, autophagy, and infectious disease-related immune signaling further support a model where chronic inflammation and impaired protein clearance converge on neuronal injury. When combining all the results, it is suggested that the turquoise module captures a neuroinflammatory–lysosomal axis that underlies many features of various neurodegenerative diseases [81]. This can be seen in Figure 6.

Based on the top 15 hub genes for the turquoise module, we identified MYD88, NFKB1, IL1B, MAPK3, SRC, CTNNB1, and PTEN as the most central genes, all of which play pivotal roles in immune regulation, kinase signaling, and autophagy, as seen in Figure 7. MYD88 and NFKB1 are central regulators of NF-κB-mediated inflammation [82,83], while IL1B encodes a cytokine elevated in multiple neurodegenerative brains [84]. MAPK3 and SRC reflect dysregulated kinase activity linked to tau phosphorylation and synaptic dysfunction [85]. Meanwhile, autophagy-related genes such as BECN1 and ubiquitin connect this inflammatory network to defective protein clearance [86,87].

Moreover, via the DEA, it was determined that seven genes play a pivotal role in the different NDs. The directions of these genes are different from each other, but they are commonly upregulated, and a few are downregulated. But these genes still play an important role in how immune regulation may affect NDs. Their overall biological and neurodegenerative relevance is summarized in the Supplementary Materials (Tables S2 and S4) which the literature is further discussed in the Discussion section [53,88,89,90,91,92,93,94,95,96,97,98].

### 3.4. miRNA–Gene Regulatory Interaction of Modules Mapped Through Network Analysis

**Figure 8:**
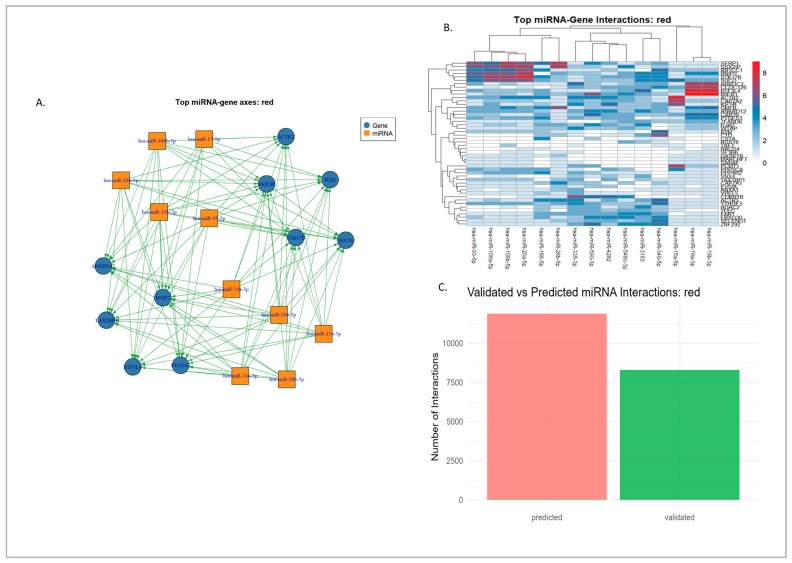
miRNA–gene interaction profiles in the red module: (**A**) Network representation of the top 10 validated axes of miRNA–gene interactions in the red module. (**B**) Heatmap of the top-ranked miRNAs and their interactions with red module genes, illustrating relative connectivity patterns. (**C**) Distribution of validated versus predicted interactions, showing the relative contribution of experimentally supported and computationally inferred edges.

Analysis of the interaction tables revealed that both the red and turquoise modules have distinct but overlapping miRNA landscapes. In the red module, 26,903 miRNA–gene interactions were identified, spanning 2132 unique miRNAs, of which 18,200 are computationally predicted and 8703 are experimentally validated. The leading miRNA hubs in the respective modules were hsa-miR-335-3p, hsa-miR-340-5p, hsa-miR-106b-5p, hsa-miR-19b-3p, hsa-miR-20a-5p, hsa-miR-590-3p, hsa-miR-19a-3p, hsa-miR-186-5p, hsa-miR-3163, and hsa-miR-548c-3p. Several of these, particularly miR-106b, miR-19a/b, and miR-20a, showed a balanced distribution between validated and predicted interactions, whereas others, such as miR-3163 and miR-548c-3p, were largely prediction-driven, as seen in Figure 8. All of this can be seen in Datasets S2 and S4. This indicates that the red module miRNA space contains both well-studied regulators and poorly characterized candidates.

**Figure 9:**
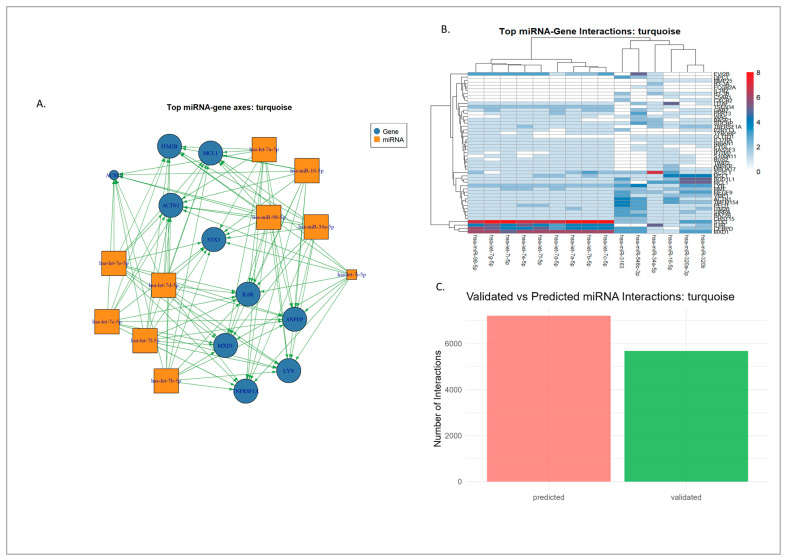
miRNA–gene interaction profiles in the turquoise module: (**A**) Network representation of the top 10 validated axes of miRNA–gene interactions in the turquoise module. (**B**) Heatmap of the top-ranked miRNAs and their interactions with turquoise module genes, illustrating relative connectivity patterns. (**C**) Distribution of validated versus predicted interactions, showing the relative contribution of experimentally supported and computationally inferred edges.

On the other hand, the turquoise module had 16,051 interactions across 2034 unique miRNAs, with 10,081 predicted and 5970 as validated. The dominant connection of miRNAs included hsa-miR-548c-3p, hsa-let-7e-5p, hsa-let-7b-5p, hsa-let-7a-5p, hsa-miR-34a-5p, hsa-miR-3163, hsa-let-7i-5p, hsa-let-7g-5p, hsa-miR-98-5p, and hsa-let-7f-5p, as seen Figure 9. Unlike the red module, the turquoise module featured multiple let-7 family members and miR-34a-5p among its top hubs, most of which were supported by substantial numbers of validated interactions, as seen in Datasets S3 and S5.

#### 3.4.1. Experimentally Validated miRNA–Gene Regulatory Networks

The experimentally validated miRNA–gene interaction networks revealed distinct regulatory patterns in both the red and turquoise modules. In the red module, several miRNAs, such as miR-34a-5p, miR-335-3p, miR-181b-5p, miR-30a-5p, and miR-26a-5p, showed strong and consistent association with their target genes as shown in Dataset S1. These miRNAs are widely recognized as regulators of cellular homeostasis, neuronal survival, and stress response. For example, mir-34a-5p, a well-established downstream effector of p53, has been linked to apoptosis regulation and mitochondrial stability in neurons. Similarly, miR-181b-5p has been reported to influence neuroinflammation and synaptic plasticity, while miR-26a-5p and miR-335-3p are known to modulate cytoskeletal organization and neurotrophic signaling.

The major hub genes within the red module, including KRAS, ACTR2, CAPZA1, and PPP2CA, were all found to interact with these several validated miRNAs. KRAS was primarily regulated by the tumor-suppressive miRNAs let-7a-5p and miR-143-3p, which are known to control MAPK signaling pathways that influence both cell proliferation and neuronal survival. In the context of neurodegeneration, this regulatory relationship may serve to balance neuronal growth signals and prevent stress-induced apoptosis. ACTR2, a component of the actin-related protein complex that governs actin nucleation, was targeted by miR-30a-5p and miR-335-3p, suggesting that these miRNAs could influence axonal remodeling and synaptic structure, processes that are often impaired in diseases such as AD and PD [71,72]. CAPZA1, another actin-associated gene involved in filament capping, was regulated by miR-26a-5p, pointing to miRNA control over cytoskeletal rearrangements that maintain neuronal morphology and transport [79,80]. Finally, PPP2CA, which encodes the catalytic subunit of protein phosphatase 2A, was targeted by miR-181b-5p. Since PP2A is a critical enzyme in tau dephosphorylation, this miRNA–gene interaction suggests a potential regulatory link to the pathological accumulation of hyperphosphorylated tau in neurodegenerative conditions [69].

In the turquoise module, validated miRNAs, such as miR-34a-5p, miR-18a-5p, miR-24-3p, miR-877-5p, and miR-423-5p, formed a robust regulatory network around several key hub genes, as seen in Datasets S1 and S4, including PTEN, HIF1A, and ACTB. PTEN was suppressed by well-known oncogenic miRNAs, such as miR-21-5p, miR-22-3p, and miR-214-3p, which are potentially involved in the activation of the PI3K/AKT signaling pathway. Although this pathway supports neuronal survival under acute stress, its prolonged activation can lead to synaptic dysfunction and aberrant protein aggregation, both of which are implicated in neurodegenerative progression [91,96]. HIF1A, a transcription factor that mediates the hypoxic response and mitochondrial metabolism, was targeted by miR-18a-5p and miR-24-3p, which might contribute to neuronal adaptation and oxidative stress and hypoxia. ACTB (β-actin), a critical cytoskeletal protein, was regulated by miR-877-5p. This miRNA could be associated with the control of neuronal shape, vesicular transport, and dendritic stability. Collectively, these experimentally validated miRNA–gene networks define core regulatory frameworks which may reflect the coordination of neuronal survival, cytoskeletal maintenance, and metabolic adaptation, all of which are fundamental to the pathophysiology of NDs.

#### 3.4.2. Hypothetical Axes from Computationally Predicted Interactions

The inclusion of computationally predicted interactions expanded the scope of the miRNA–gene networks and uncovered several potential regulatory pathways that may complement the experimentally validated findings. In the red module, predicted miRNAs, such as miR-495-3p, miR-338-5p, miR-498-5p, and miR-5692a, were identified as regulators of important hub genes, including KRAS, ACTR2, CAPZA1, and PPP2CA, as well as other genes such as RAB14 and ATP6V1E1. Many of these predicted miRNAs are linked to neuronal differentiation, mitochondrial regulation, and vesicular transport, suggesting a strong connection between the red module network and neuronal energy metabolism. For example, miR-338-5p has been associated with mitochondrial function and axonal energy distribution, while miR-495-3p was determined to possibly interact in regulating BDNF signaling, which influences neuronal survival and plasticity. The prediction that these miRNAs target genes involved in cytoskeletal and signaling functions indicates that they may contribute to maintaining structural integrity and intracellular communication in neurons.

In the turquoise module, the computational predictions revealed a concentration of miRNAs from the miR-548-family, including miR-548aj-3p, miR-548x-3p, and miR-548ah-3p, which were linked to hub genes PTEN, HIF1A, and ACTB. Although the miR-548-3p family remains poorly characterized, emerging studies suggest that these miRNAs may play a role in regulating immune responses, autophagy, and stress adaptation—processes that are critical in the progression of NDs. Other predicted miRNAs, including miR-200-3p and miR-204-3p, were associated with oxidative stress regulation and endoplasmic reticulum homeostasis. These predicted interactions suggest that the turquoise module possibly integrates both metabolic and cytoskeletal adaptation mechanisms, potentially modulating cellular responses to chronic neuronal stress. Overall, computational predictions complement these experimentally validated findings by identifying additional candidate miRNAs that may fine-tune key molecular processes relevant to neurodegeneration.

#### 3.4.3. Synthesis and Prioritized Hub Regulatory Axes

**Figure 10:**
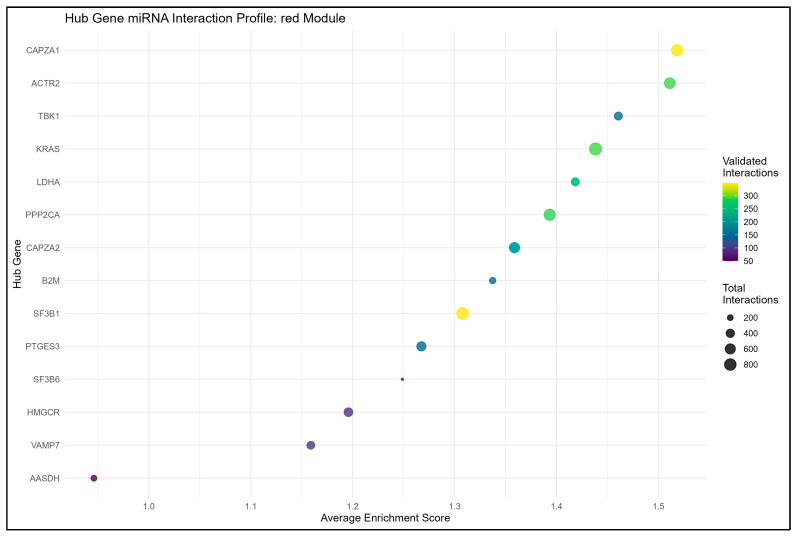
Hub gene–miRNA interaction profile for the red module.

Integration of the experimentally validated miRNA–gene interactions identified several hub-centered regulatory networks that are likely to play central role in neuronal stability and stress adaptation. In the red module, validated interactions converge strongly on KRAS, ACTR2, CAPZA1, and PPP2CA, together forming a regulatory cluster that governs cytoskeletal organization, phosphate activity, and intracellular signaling, as seen in Figure 10. The KRAS-let-7a-5p and KRAS-miR-143-3p axes were associated with the modulation of MAPK signaling, a pathway linked to neuronal survival and oxidative stress response. ACTR2 and CAPZA1/2, both actin-related genes, were targeted by miR-30a-5p, miR-335-3p, and miR-26a-5p, suggesting that these miRNAs contribute to the maintenance of axonal architecture. Also, these hub genes were suggested to be associated with reduced repression of the miR-29 family members, potentially linking to synaptic vulnerability [92] PPP2CA, which is regulated by miR-133a, miR-125b, and miR-181b-5p, is potentially linked to phosphorylation and synaptic signaling, while HMGCR, TBK1, and B2M may reflect an implicated metabolic dysregulation, impaired autophagy, and immune activation [78,79,88].

**Figure 11:**
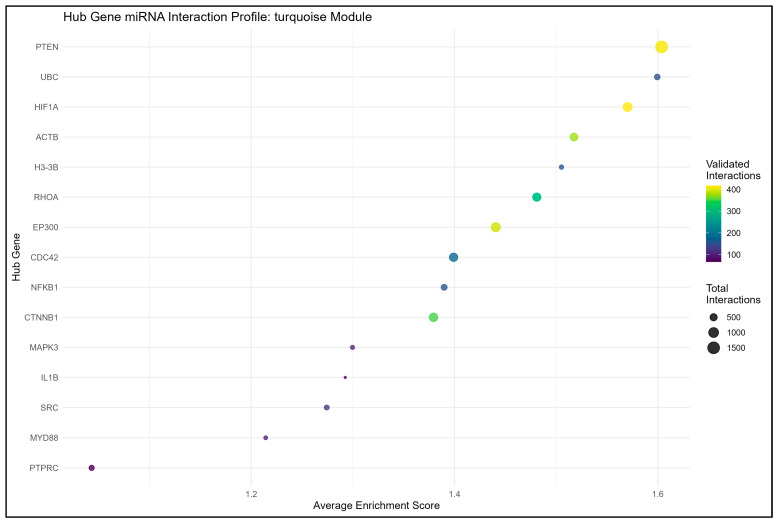
Hub gene–miRNA interaction network of the turquoise module.

The turquoise module highlighted complementary processes. ACTB and EP300 are linked to miR-132 and miR-22, which highlights a possible mechanism of cytoskeletal integrity and chromatin remodeling. PTEN, which is linked to miR-19b, miR-21-5p, miR-22-3p, and miR-214-3p, is reflected in the impairment of neuronal survival signals [91,93], while HIF1A, RHOA, and other hub genes, including IL1B, NFKB1, and SRC, and PTPRC, raise the possibility of metabolic stress and chronic inflammation due to their being linked with miR-146a and miR-223 [90,94]. The validated interactions of these hub genes could be seen in Figure 11.

It is important to note that while these integrated miRNA–gene networks reveal biologically coherent relationships, many of the interactions available in public databases are derived from studies in cell lines or animal models. Their relevance to human NDs should be interpreted with caution. These findings represent hypotheses that highlight promising regulatory axes for further validation in disease-relevant neuronal or glial models.

### 3.5. In Silico Validation of Hub Genes with GWAS Traits

Out of the 30 hub genes, 29 of the hub genes had results in the Open Target Platform; the gene SPTCL1 showed no results in the database. Among the 29 hub genes, most showed moderate-to-strong evidence of association, with a global score of ≥0.3, with at least one disease, while some hub genes, such as PTEN, KRAS, PPP2CA, TBK1, and CTNNB1, displayed a particularly high global score, i.e., is around ≥0.8. Notably, a subset of these genes, including TBK1 and HMGCR, exhibited strong association with neurodegenerative conditions, especially AD and ALS, while also being linked to metabolic and immune-related traits, which can be seen in Dataset S6. These patterns highlight the potential shared molecular mechanism connecting neurodegeneration with systemic pathways such as lipid metabolism, inflammation, and cellular signaling [70,73,74].

At the module level, the majority of the associations were contributed by the turquoise module, which encompassed genes broadly linked to cellular regulation and signaling; whereas the red module encompasses genes with more neuro-specific and metabolic associations, such as TBK1 and HMGCR. These findings suggest that while both modules capture biologically relevant gene programs, they may also represent pathways more directly tied to neurodegenerative processes and immune-metabolic interactions. Overall, the Open Targets Platform serves to validate and support the clinical and biological relevance of the identified hub genes and to provide additional evidence that the network-derived modules capture disease-relevant molecular signatures.

## 4. Discussion

This study relies on the use of WGCNA to organize thousands of genes into a functional co-expression network, revealing stable modules shared across AD, ALS, HD, and PD. By integrating blood RNA-Seq datasets, we identified conserved co-expression modules that reflect systemic molecular signatures of NDs. Two modules emerged as consistently preserved, the red module and the turquoise module. The red module is enriched for cytoskeletal and metabolic regulation, while the turquoise module is enriched for immune and proteostatic pathways. Both are limited in terms of sample size and PD-specific alteration in mitochondrial or synaptic regulation [49,50]. While these in silico or computational findings highlight robust co-expression signatures, experimental validation will be essential to confirm their biological relevance and therapeutic potential.

### 4.1. Hub Genes as Central Regulators of Pathways in Neurodegeneration

Preserved modules are anchored by hub genes that serve as a regulatory bottleneck within shared molecular pathways. In the red module, cytoskeletal regulators such as ACTR2 and CAPZA1/2 coordinate the filament turnover, a process that is essential for axonal transport and synaptic vesicle trafficking, wherein the disruption of these hubs may impair vesicle mobility, destabilize dendritic spines, and weaken synaptic communication [96]. These changes are said to be hallmarks of AD, wherein actin destabilization coincides with tau hyperphosphorylation, and also of ALS, wherein impaired axonal transport may accelerate neuronal death [97,98]. Their vulnerability under oxidative stress and excitotoxic conditions further emphasizes cytoskeletal hubs as shared points of failure across neurodegeneration.

Another core hub gene of the red module was HMGCR, which regulates cholesterol biosynthesis. Cholesterol is not only a metabolic structure but also a critical membrane for fluidity, vesicle fusion, and receptor clustering [58,70,99]. The possible dysregulation of HMGCR compromises neuronal signaling and has been said to be linked to amyloid precursor protein (APP) processing, which favors amyloidogenic cleavage and β-amyloid accumulation in AD [100]. However, the impact of cholesterol dysregulation extends beyond AD. In ALS, disturbances in lipid metabolism have been linked to energy imbalance and neuronal vulnerability; while in PD, cholesterol handling intersects with α-synuclein aggregation. This reflects that hub gene regulating controls are not disease-specific but act as a systemic regulator of neuronal resilience. This could possibly explain why lipid regulation is a systemic vulnerability bridging cardiovascular risk and neurodegeneration [101].

On the other hand, the hub genes on the turquoise module, such as MYD88, NFKB1, and IL1B, are driving immune and survival regulators. These hubs are said to mobilize microglia and astrocytes against misfolded proteins and cellular debris [72,73]. While protective early, sustained activation drives chronic neuroinflammation, oxidative stress, and cytokine release, worsening neuronal death [102]. Furthermore, hubs like PTEN and EP300 are important; they regulate survival and transcriptional adaptation. PTEN acts as a brake on PI3K/AKT signaling, tipping the balance towards apoptosis when overstimulated. In the context of neurodegeneration, elevated PTEN activity potentially reflects the capacity of neurons to withstand oxidative or proteotoxic stress [86,92,102]. EP300 regulates histone acetylation and stress-induced transcription. Dysregulation of these hubs may lead to the possibility of adaptive stress responses into progressive and maladaptive cascades [101].

Interestingly, despite the enrichment for stress-response and proteostasis pathways in the turquoise module, we did not detect classical heat shock proteins or HSPs, just like HSP70 and HSP90, among the preserved hubs. These molecular chaperones are well-recognized regulators of protein folding, the suppression of toxic aggregates, and neuroprotection in AD, PD, HD, and ALS. Their absence in our preserved modules may reflect tissue-specific regulation since HSP expression in strongly induced in neuronal tissues under stress but is less consistently detectable in blood samples [97,102]. Nonetheless, HSPs remain a crucial axis of proteostasis, and their dysregulation is central to neurodegeneration, which is why there is a need for further study comparing blood and brain samples in transcriptomic analysis [103].

#### Integration of Hub Genes with Genome-Wide Association Studies

Several hub genes identified in the preserved modules are said to be associated with loci that are found in NDs through GWAS. For instance, variants within or near PTEN, HMGCR, and NFKB1 have been linked to AD and PD susceptibility [82,91,104,105], while TBK1, which is a key immune and autophagy regulator, is said to be linked in ALS, wherein loss of function mutations could possibly disrupt protein clearance pathways [73,74]. The convergence of these genes across our co-expression modules and in silico validation through GWAS suggest that systemic molecular alteration in blood reflect, at least in part, can potentially influence mechanisms contributing to neurodegeneration.

However, this convergence should be interpreted cautiously. GWAS association reflects genetic predisposition, whereas our transcriptomic modules represent dynamic expression states that can result from, or respond to, pathology. The observed overlap therefore highlights potential mechanistic intersections rather than direct causation. Future studies integrating genotype expression (eQTL) data from matched blood and brain tissues will be needed to clarify whether these hub genes mediate genetic risk or reflect downstream adaptation to neurodegenerative processes.

### 4.2. miRNA–mRNA Regulatory Collapse and Pathway Disinhibition

miRNAs serve as molecular governors that keep hub gene activity in check or within homeostatic bounds. In the red module, mi-29 miR-133a, and miR-125b are said to maintain cytoskeletal homeostasis by regulating genes involved in axonal transport and dendritic stability [106]. In disease results or statistics, however, these miRNAs are consistently downregulated, which allows for genes like ACTR2 and PPP2CA not to undergo repression, which potentially reflects the result of having a hyperactive cytoskeletal remodeling process that paradoxically weakens neuronal structure instead of reinforcing it. This dysregulation is evident in AD, where miR-29 and miR-125b reduction possibly correlates with tau pathology, but it is equally relevant in ALS and Huntington’s, where cytoskeletal collapse drives early synaptic failure [107].

In the turquoise module, miRNAs such as miR-132, miR-22, miR-19b, and miR-146a normally function as critical checkpoints that restrict immune and survival pathways, preventing them from overshooting [108]. When these miRNAs are downregulated in diseases, hub genes, including IL1B, PTEN, and EP300, may become unrestrained, which leads to the possibility of driving chronic and maladaptive activation [109].

On the immune side, miR146a and miR223 are thought to function as feedback inhibitors of MYD88-dependent toll-like receptor signaling, suppressing excessive NF-kB activation. Their loss allows even minor triggers, such as amyloid-β in AD or α-synuclein in PD, to escalate into persistent inflammation [110,111]. This model suggests that instead of mounting a temporary defense, microglia and astrocytes remain locked into a pro-inflammatory state, releasing cytokines and reactive oxygen species that damage nearby neurons. Similarly, reduced levels of miR-132 and miR-22 appear to impair the repression of IL1B, potentially further amplifying inflammatory cascades. Without these miRNA brakes, inflammation may possibly lose its self-limiting nature, transforming itself from a protective mechanism into a driver of pathology for the onset of diseases [107,110].

Survival signaling also appears to be destabilized by miRNA collapse. miR19-b, which normally inhibits PTEN, safeguards PIR3K/AKT signaling and supports neuronal resistance to stress. Loss of this repression likely raises PTEN activity, potentially tipping the balance toward apoptosis when neurons encounter oxidative or proteotoxic stress [96]. Similarly, miR-22 normally tempers EP300, ensuring that stress-induced chromatin remodeling enhances resilience rather than maladaptation. Its downregulation is said to be associated with maladaptive transcriptional programs that weaken synaptic plasticity and memory functions [112,113,114].

### 4.3. Clinical and Translational Relevance of Hub Genes and miRNAs

One unique strength of this study lies in its translational potential. By focusing on blood-derived transcriptomes, we captured molecular changes that are clinically accessible, opening the possibility of developing circulating biomarkers for neurodegenerative diseases. Unlike brain tissue, which is inaccessible during life, blood can be repeatedly sampled to monitor disease onset, progression, and treatment response. The consistent identification of hub gene–miRNA axes in peripheral blood samples, therefore, raises the possibility that these signatures have the potential to provide early-warning indicators of neurodegeneration. For instance, downregulation of miR-29 or miR-132 in the blood might hint at signal early synaptic instability or inflammatory priming, potentially enabling early intervention before irreversible neuronal loss occurs—a hypothesis that, if validated in prospective clinical cohorts, could enable earlier intervention before irreversible neuronal loss occurs [110,111,112].

Equally significant is the hypothetical therapeutic potential embedded in the hub genes themselves. Several hubs intersect with established pharmacological landscapes, raising the possibility or prompting of hypothesizing drug repurposing. In the red module, HMGCR stands out as it is the target of statins, which are widely prescribed for cardiovascular diseases. Epidemiological studies have suggested that those statins may reduce AD risk, though the results differ from time to time, which leads to inconsistency [115,116,117]. Identification of HMGCR as a hub gene strengthens the mechanistic plausibility of cholesterol metabolism and impacts neuronal resilience, but it does not yet confirm the efficacy of statins for neurodegeneration. Similarly, PPP2CA, a phosphate that regulates tau dephosphorylation, has direct relevance to AD’s pathology, and experimental PP2A activators are already being explored in preclinical settings [69,117]. These examples highlight that systemic metabolic regulators are plausible candidates for further neuroprotective investigations.

In the turquoise module, several hubs connect to immune and survival signaling, many of which are already druggable. MYD88 and NFKB1, central to toll-like receptor and NF-kB signaling, are therapeutic targets in cancer and autoimmunity, with inhibitors and antibodies that could be repurposed for NDs [82,83]. IL1B offers an especially tangible example: its blockade by anakinra or an IL-1 receptor antagonist is already FDA-approved for inflammatory disorders, theoretically enabling its rapid translation application, with its efficacy still to be demonstrated in neurodegenerative patients [89,90,118,119]. Other hubs, such as EP300 and PTEN, connect trials, while PTEN modulation is under exploration for regenerative therapies [88,96,112].

When integrated with miRNA regulation, the translation picture becomes even richer. miRNAs such as miR-29, miR-132, miR-146a, and let-7 family members not only emerge as systemic biomarkers but also as potential therapeutic agents themselves. The use of miRNA mimics or antagomirs to restore post-transcriptional control is being actively evaluated in cancer and cardiovascular diseases, and our findings suggest a similar potential in neurodegeneration. Importantly, the integration of hub-directed therapies with miRNA-based approaches may provide synergistic benefits, just like, for example, NF-kB activity, while restoring miR-146a could simultaneously suppress immune overactivation and reinstate regulatory balance [120,121,122,123,124,125,126].

However, it is crucial to acknowledge several key limitations of this study. The biomarker signatures are promising, but they need to be validated in large patient groups. The therapeutic connections we suggest, such as statins or anakinra for brain disease, are hypothesis based on our data and findings but have not yet been proven in application. Future work must test these ideas directly in experimental models and clinical trials. In summary, our study provides a valuable map for future research, highlighting the most promising paths for developing diagnostics and treatments.

### 4.4. Blood vs. Brain Transcriptomic Signatures

Although the brain is the principal site of neurodegeneration, increasing evidence suggests that blood transcriptomes can partially reflect central nervous system (CNS) pathology through shared immune and metabolic signaling pathways [118]. Neurodegenerative diseases involve not only neuron-intrinsic degeneration but also systemic inflammation, mitochondrial stress, and lipid dysregulation—processes that are also evident in circulating leukocytes. Therefore, blood provides a clinically accessible window into systemic aspects of NDs, even if it cannot capture region-specific neuronal events [119,120].

In our analysis, the red and turquoise modules were consistently preserved across AD, ALS, HD, and PD, reflecting broad systemic alterations. However, the turquoise module showed weaker preservation in PD, possibly due to disease-specific mitochondrial or dopaminergic mechanism or low sample counts and technical issues among datasets. This contrast underscores that blood captures overlapping but not identical processes compared with the brain, while inflammatory and metabolic responses are mirrored peripherally, neuronal and synaptic pathways remain largely CNS-restricted [37,127,128,129,130,131]. Additionally, the absence of classical heat shocks among preserved blood modules supports this distinction; HSP expression is robustly induced in neurons under proteotoxic stress but modestly in blood cells [103,104].

Together, these comparisons highlight that blood and brain transcriptomes represent complementary layers of neurodegenerative biology. Blood reflects systemic immune and metabolic dysregulation, which may influence or respond to CNS pathology; whereas brain tissue reveals cell-intrinsic neuronal and glial mechanisms. Integrating both sources through matched transcriptomic or proteomic analysis will be essential to determine which molecular signatures faithfully mirror central processes and to develop blood-based biomarkers with verified neurobiological relevance.

### 4.5. Limitations and Future Recommendations

Although this study provides valuable insight into conserved molecular modules and regulatory networks across major neurodegenerative diseases, several limitations should be acknowledged to guide interpretation and future research. First, the datasets analyzed were derived exclusively from blood RNA-seq profiles, which, while clinically accessible and reflective of systemic processes, may not fully capture brain-specific transcriptional dynamics or cell-type-based restricted pathways involved in neuronal degeneration. Validation using post-mortem brain tissue, single cell transcriptomics, and proteomic datasets will be essential to determine whether the identified blood signatures truly parallel central nervous system alterations.

Second, our study relied on publicly available datasets with inherent variability in study design and biological heterogeneity. These include difference is sequencing platforms, sample sizes, clinical inclusion criteria, and metadata completeness, and some datasets lacked covariates such as age or sex. Meanwhile, in the AD cohort, mild cognitive impairment (MCI) samples were grouped with AD to maintain statistical power and reflect the biological continuum between the two conditions. Such variability is an unavoidable feature of secondary transcriptomic analyses and does not compromise the analytical framework; rather, it underscores the importance of the harmonization strategies, such as consistent preprocessing, covariate correction, and normalization, applied in this study. Nonetheless, these factors should be considered when generalizing our findings, as subtle cohort- or platform-specific effects may influence gene co-expression strength.

Third, the interpretation of miRNA–mRNA interactions and hub gene centrality is based on transcript-level association and in silico predictions, which should be viewed as hypothesis-generating. Experimental validation, such as via luciferase reporter assays, CRIPSRT perturbation, miRNA mimic, or inhibitor experiments, is highly necessary to confirm causality and refine mechanistic understanding. Additionally, integrating epigenetic, proteomic, and metabolomic layers could strengthen the biological inference of these transcription modules and reveal upstream regulatory hierarchies. Moreover, considering that the interactions of most databases are derived from cell lines or animal-based studies, validation in terms of patient-derived blood, glial, and neuronal information is needed in order to capture the disease-specific biology that occurs in NDs.

Fourth, from a translational standpoint, the identification of hub genes such as HMGCR, PTEN, and IL1B highlights potentially druggable targets, but clinical applications require careful experimental and longitudinal validation. Future works should integrate co-expression and GWAS-derived evidence with expression-quantitative trait locus (eQTL) and longitudinal clinical data to clarify whether blood-based molecular alterations represent causal risk factors or secondary consequences of neurodegeneration. Comparative analyses between blood and brain datasets, as well as NDs and non-NDs, will further delineate systemic versus disease-specific pathways.

In summary, while this study is subject to the inherent constraints of secondary data analysis, it provides a robust, reproducible network-level map of molecular convergence across NDs by acknowledging dataset variability and emphasizing the need for refining blood-based biomarkers and identifying shared therapeutic targets in complex NDs.

## 5. Conclusions

This study shows that different NDs, including AD, ALS, HD, and PD, share common molecular patterns that can be detected in blood transcriptomes. The preserved gene networks we identified, particularly the red and turquoise modules, include cytoskeletal organization, cholesterol metabolism, immune activation, and protein quality control as central features of disease. Within these networks, hub genes such as ACTR2, HMGCR, MYD88, and EP300 were found to be key regulators, linking several disease mechanisms together. We also found that important microRNAs, including miR-29, miR-132, and miR-146a, were consistently reduced, potentially leading to a loss of control over these hub genes and allowing harmful processes to continue unchecked. Furthermore, the absence of classical heat shock protein in the preserved blood modules further underscores the tissue-specific transcriptional regulation between the blood and the brain.

The clinical importance of these findings lies in their accessibility. Because they are detectable in blood, these molecular signatures could serve as minimally invasive biomarkers to assist in early disease diagnoses, tracking progression, or guiding patient care. At the same time, the hub genes we identified overlap with known drug targets, potentially making them strong candidates for repurposing therapies that already exist. The combination of hub gene targets and microRNA regulation also highlights a dual strategy for treatment: restoring balance through drugs that target central pathways while using microRNA-based therapies to re-establish post-transcriptional control. Taken together, our results provide both a clearer understanding of shared disease mechanisms and a hypothesis-gathering direction for developing new diagnostic and therapeutic approaches in neurodegenerative disorders. However, as these results are derived from the in-silico analysis of heterogenous public datasets, experimental and multi-omic validation will be required to confirm their mechanistic and clinical significance. Overall, this study provides a foundational framework for exploring shared molecular pathways and translational targets across NDs.

## Figures and Tables

**Table 1:** Summary of neurodegenerative disease RNA-Seq data used in study.

Accession No.	GSE51799 [30]	GSE234297 [31]	GSE165082 [32]	GSE249477 [33]
Condition	Huntington’s Disease	Amyotrophic Lateral Sclerosis (ALS)	Parkinson’s Disease	Alzheimer’s Disease
Type	Expression Profiling by High Throughput Sequencing			
Source	Blood Samples			
No. of Samples	124	132	26	62

**Table 2:** Z summary thresholds for module preservation.

Z Summary Score	Interpretation
Z summary > 10	Strong evidence of preservation
2 < Z summary ≤ 10	Moderate evidence of preservation
Z summary ≤ 2	No Evidence of Preservation

**Table 4:** Preservation patterns of neurodegenerative disorders in different modules.

Module	Preservation Pattern Across HD, PD, and ALS
Red	Preserved across all diseases
Turquoise	Preserved in HD, ALS; disrupted in PD
Blue	Stable in HD, ALS; weaker in PD
Green	Stable in HD, ALS; disrupted in PD
Yellow	Variable across diseases
Brown	Weak in HD; modest in ALS
Gold	Disrupted in PD
Pink	Variable across diseases

## Data Availability

The transcriptomic datasets analyzed in this study are publicly available from the Gene Expression Omnibus (GEO) database (https://www.ncbi.nlm.nih.gov/geo/ accessed on 10 July 2025). The datasets include Huntington’s disease (GSE51799), Amyotrophic Lateral Sclerosis (ALS; GSE234297), Parkinson’s disease (GSE165082), and Alzheimer’s disease (GSE249477).

## Supplementary Materials

The following supporting information can be downloaded at https://www.mdpi.com/article/10.3390/genes16101178/s1: Figure S1: Dispersion Estimates of RNA-Seq Data in Neurodegenerative Diseases; Figure S2: Scale-Free Topology Model Fit Across Neurodegenerative Disease Networks (Individual); Table S1: Summary of Dataset Demographic and Disease Classification; Table S2: Cross-Disease Differential Expression of Candidate Genes in Neurodegenerative Disorders; Table S3: Biological Function and Neurodegenerative Relevance of Red Module Hub Genes and Direction; Table S4: Biological Function and Neurodegenerative Relevance of Turquoise Module Hub Genes and Direction; Dataset S1: Summary of Preserved Modules in Different Permutations; Dataset S2: miRNA Regulation Red; Dataset S3: miRNA Regulation Turquoise; Dataset S4: Hub-Gene and miRNA Interaction Red; Dataset S5: Hub-Gene and miRNA Interaction Turquoise; Dataset S6: GWAS of Different Hub Genes.

## Abbreviations

AD	Alzheimer’s Disease
ALS	Amyotrophic Lateral Sclerosis
HD	Huntington’s Disease
ND	Neurodegenerative Diseases
PD	Parkinson’s Disease
WGCNA	Weighted Gene Co-Expression Network Analysis
