# Supplementary material for: Conserved Blood Transcriptome Patterns Highlight microRNA and Hub Gene Drivers of Neurodegeneration
Source: Genes (Basel). 2025 Oct 10;16(10):1178. doi: 10.3390/genes16101178 (PMC12562450; doi:10.3390/genes16101178)
Supplement: Supplementary file 1 [file genes-16-01178-s001.zip › Supplementary Tables.pdf]

**Table S1**. Summary of Dataset Demographic and Disease Classification

Legend: A comparative summary of the four neurodegenerative disease datasets, detailing total sample size, the distribution of cases and controls, and the availability of key demographic variables.

| Dataset | Total Samples | Disease Group Count | Control Group | Age Information | Sex Information |
|---|---|---|---|---|---|
| **AD** | 62 | AD (21)<br>MCI (20) | Normal (21) | Available<br>Ranging from<br>73- 93 | Available<br>29 Males<br>33 Females |
| **ALS** | 132 | ALS (86) | Normal (46) | Not Available | Not Available |
| **HD** | 124 | HD (91) | Normal (33) | Available<br>Ranging from<br>22-75 | Available<br>56 Males<br>68 Females |
| **PD** | 26 | PD (12) | Normal (14) | Not Available | Not Available) |

**Table S2**. Cross-Disease Differential Expression of Candidate Genes in Neurodegenerative Disorders

Legend: This table summarizes the direction and statistical significance of differential gene expression for candidate genes across AD, PD, HD, and ALS along with cross-disease consensus trends and classification tier.

| Gene | AD | PD | HD | ALS | Cross-Disease Consensus | Tier |
|---|---|---|---|---|---|---|
| **HMGCR** | UP (p=0.199, LFC=0.660) | | | | Nominally up in AD | 2 |
| **ACTR2** | UP (p=0.483, LFC=0.507) | | | | Nominally up in AD | 2 |
| **TBK1** | UP (p=0.384, LFC=0.561) | | | | Nominally up in AD | 2 |
| **SF3B1** | UP (p=0.459, LFC=0.523) | | | | Nominally up in AD | 2 |
| **PTPRC** | UP (p=0.260, LFC=0.634) | | | UP (p=0.969, LFC=0.287) | PD, ALS | 3 |
| **PTEN** | UP (p=0.861, LFC=0.326) | | | UP (p=0.965, LFC=0.316) | PD, ALS | 3 |
| **CAPZA2** | UP (p=0.567, LFC=0.460) | | | | Nominally up in AD | 2 |
| **B2M** | UP (p=0.893, LFC=0.270) | | | | Nominally up in AD | 2 |
| **CAPZA1** | UP (p=0.878, LFC=0.281) | | | | Nominally up in AD | 2 |
| **EP300** | UP (p=0.680, LFC=0.410) | | | | Nominally up in AD | 2 |
| **IL1B** | DOWN (p=0.830, LFC=-0.350) | | | UP (p=0.957, LFC=0.337) | Mixed direction | 3 |
| **HIF1A** | | | | UP (p=0.659, LFC=0.449) | Nominally up in ALS | 2 |
| **UBC** | | | UP (p=0.519, LFC=0.490) | | Nominally up in HD | 2 |
| **PTGES3** | DOWN (p=0.980, LFC=-0.254) | | | | Nominally down in AD | 2 |
| **NFKB1** | DOWN (p=0.976, LFC=-0.251) | | | | Nominally down in AD | 2 |
| **MAPK3** | DOWN (p=0.821, LFC=-0.383) | | | | Nominally down in AD | 2 |

**Table S3.** Biological Function and Neurodegenerative Relevance of Red Module Hub Genes and Direction

Legend: This table lists hub genes identified in the red co-expresison module, describing each gene;s biological function and its reported roles or associations in neurodegenerative processes (e.g., synaptic remodeling, cholesterol metabolism, and immune signaling)

| Gene | Biological Relevance | Relevance to Neurodegenerative |
|---|---|---|
| **HMGCR** | Key enzyme in cholesterol and isoprenoid production [63] | Alters cholesterol balance; may affect amyloid processing (AD) and dopamine neuron survival (PD) [70] |
| **ACTR2** | Controls actin remodeling through Arp2/3 complex [63] | Disrupted actin remodeling impairs synapses and autophagy (AD, PD)[71], [72] |
| **TBK1** | Regulates immune signaling, autophagy and mitophagy [63]. | Mutations cause ALS/FTD; altered activity drives neuroinflammation and defective clearance [73], [74]. |
| **SF3B1** | Core spliceosome protein for RNA splicing [63] | Mutations cause ALS/FTD; altered activity drives neuroinflammation and defective clearance [75], [76] |
| **CAPZA2** | Caps actin filaments to control length and stability [63] | Regulates dendritic spine strength; disruption weakens synapses [77] |
| **B2M** | Component of MHC class I found in blood and CSF [63] | High levels linked to cognitive decline, reduced neurogenesis, and inflammation [78] |
| **CAPZA1** | Similar actin capping role as CAPZA2.[63] | Disruption destabilizes synapses and impairs neuron signaling [79], [80] |

**Table S4.** . Biological Function and Neurodegenerative Relevance of Turquoise Module Hub Genes and Direction

Legend: This table list hub genes from turquoise co-expression module, highlighting each gene's known biological function and its relevance to neurodegeneration-related mechanisms such as inflammation, proteostasis, and cellular responses

| Gene | Biological Relevance | Relevance to Neurodegenerative |
|---|---|---|
| **EP300** | Histone acetyltransferase regulating chromatin and transcription.[63] | Controls plasticity, memory, stress response; dysregulation shifts toward degeneration [88]. |
| **IL1B** | Pro-inflammatory cytokine from microglia/macrophages.[63] | Drives neuroinflammation; chronic elevation worsens AD pathology, context-dependent roles [89], [90] |
| **HIF1A** | Transcription factor for oxygen response, angiogenesis, metabolism.[63] | Protective under hypoxia; sustained activation adds stress, linked to ALS and AD [91] |
| **UBC** | Source of ubiquitin for protein degradation [63] | Proteostasis failure in AD, PD, HD; altered expression reflects stress response [92], [93] |
| **PTPRC** | Immune cell marker (CD45) for microglia activation.[63] | Elevated in neuroinflammation; persistent activation drives AD, PD, ALS progression [94], [95] |
| **PTEN** | Regulates PI3K–AKT pathway, survival, synapse size, regeneration.[63] | Overactivation increases vulnerability; partial inhibition supports resilience [96] |
| **PTGES3** | HSP90 co-chaperone (p23), stabilizes receptors, stress response.[63] | Dysregulation disrupts folding and stress granules; implicated in AD and ALS [97] |
| **NFKB1** | NF-κB transcription factor, key in inflammation and stress.[63] | Chronic overactivation drives neuroinflammation (AD, PD); controlled activation can protect [82], [98] |
