# Supplementary material for: Conserved Blood Transcriptome Patterns Highlight microRNA and Hub Gene Drivers of Neurodegeneration
Source: Genes (Basel). 2025 Oct 10;16(10):1178. doi: 10.3390/genes16101178 (PMC12562450; doi:10.3390/genes16101178)
Supplement: Supplementary file 1 [file genes-16-01178-s001.zip › Supplementary Figures.pdf]

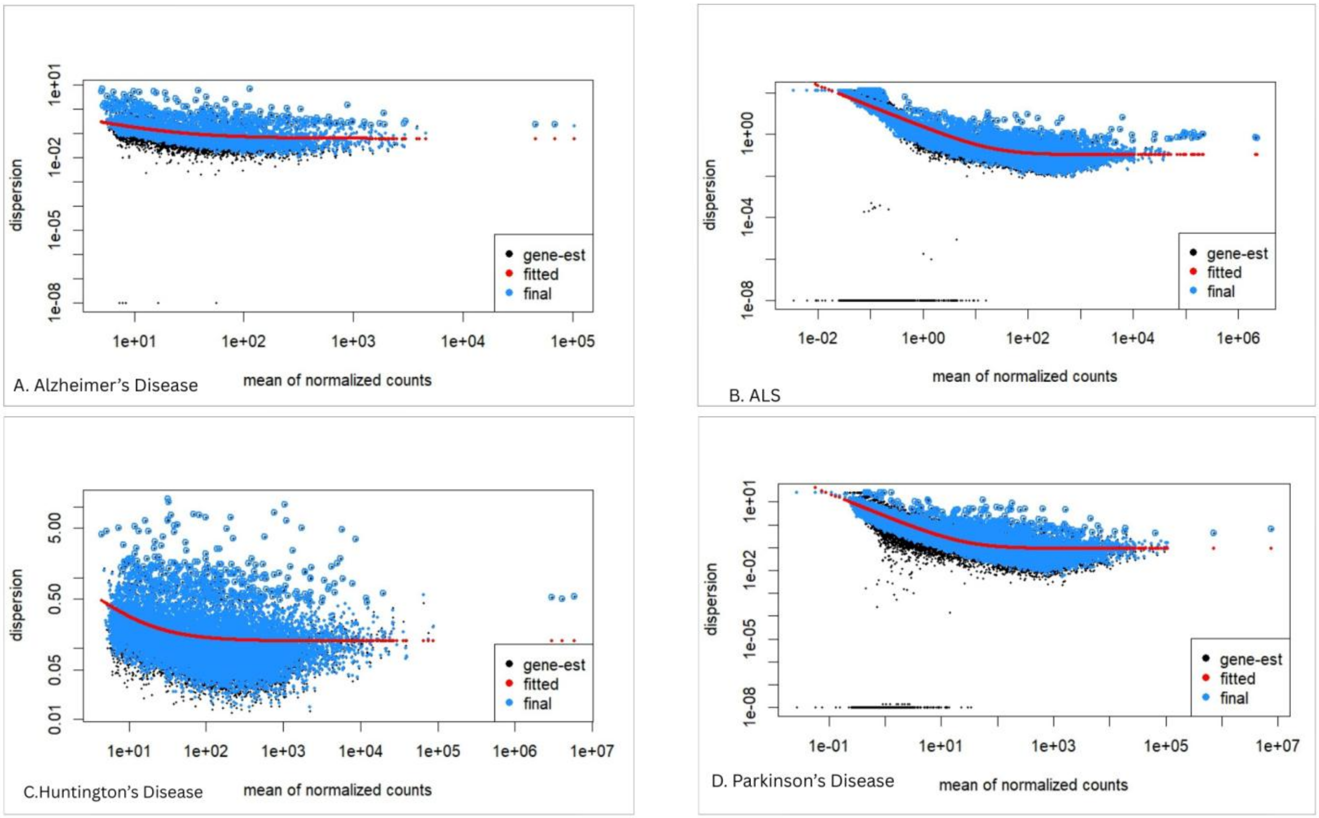

Figure S1. Dispersion Estimates of RNA-Seq Data in Neurodegenerative Diseases

Mean–dispersion plots generated from DESeq2 showing the relationship between mean normalized counts (x-axis) and dispersion estimates (y-axis) for genes in four neurodegenerative disease datasets. Each dot represents a gene: black dots indicate the gene-wise raw dispersion estimates (gene-est), the red line shows the fitted dispersion trend (fitted), and blue dots represent the final dispersion estimates after shrinkage (final). Panel A corresponds to Alzheimer's disease, Panel B to amyotrophic lateral sclerosis (ALS), Panel C to Huntington's disease, and Panel D to Parkinson's disease. These plots illustrate the variance stabilization applied by DESeq2, ensuring reliable differential expression analysis across genes with varying expression levels.

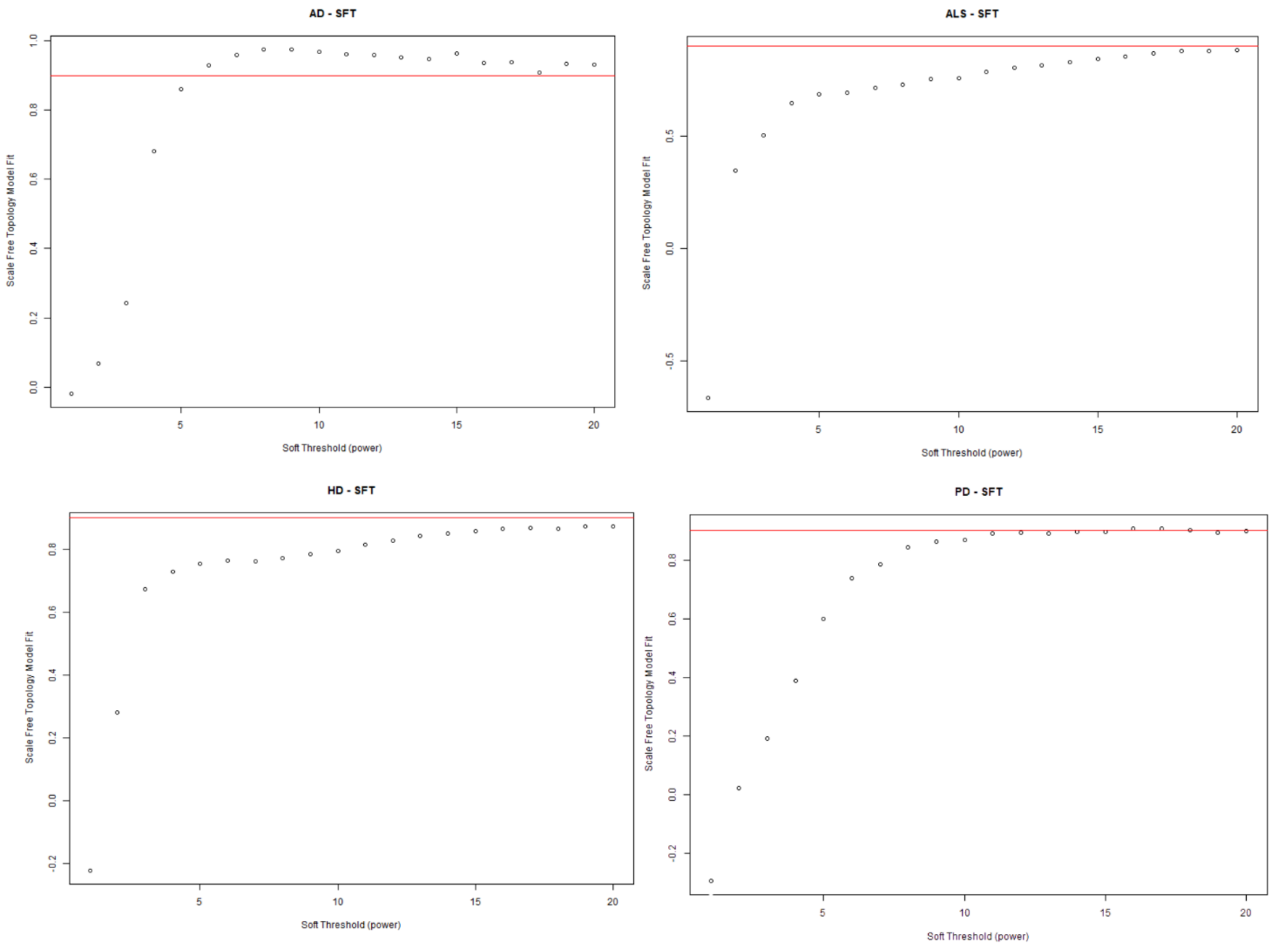

Figure S2. Scale-Free Topology Model Fit Across Neurodegenerative Disease Networks (Individual)

Plots show the scale-free topology model fit (y-axis) as a function of the soft thresholding power (x-axis) for co-expression networks constructed from samples of Alzheimer's disease (AD), amyotrophic lateral sclerosis (ALS), Huntington's disease (HD), and Parkinson's disease (PD). The horizontal red line indicates the commonly used threshold ($R^2 = 0.85$) for approximate scale-free topology. Points represent model fit values at different soft-thresholding powers (1–20), with higher values indicating a better approximation of scale-free network structure.
